# Atropine as an Antidote to Opium Poisoning

**Published:** 1868-12

**Authors:** M. S. Buttles


### Atropine as an Antidote to Opium Poisoning.

BY M. S. BUTTLES, M. D.

The following is the history of a case occurring in my practice, which shows the value of belladonna in cases of poisoning by opium:

Mrs. W., aged 38, has been troubled with retroversion, perimetritis, and severe endometritis, and has had several severe attacks of pericarditis, which have left extensive adhesions.

Had severe neuralgic pains all along the left side, for which I had been in the habit of giving her subcutaneous injections of gr. ss. morphia sulphatis.

On January 20th last, I gave her one of these hypodermic injections, which gave but slight relief; the next morning I repeated it, injecting exactly minim xv, Magendie's solution, (equal to gr. ss. morphia,) and remained in the room fifteen or twenty minutes, when she seemed a little easier, and I retired to my office down stairs; but was very soon summoned by the nurse, who stated that Mrs. W. was dying.

I found her lips purple, the respiration seven per minute, no pulse at the wrist, but one sound at the heart; pupils contracted to a fine point, frothing at the mouth, and the extremities cold.

I commenced artificial respiration (for while I was cogitating on my handiwork, she entirely stopped breathing,) which by myself and assistants was kept up for about half an hour, when I attempted to give her some strong coffee, but she could not be made to swallow. I had sent for several neighboring physicians, who were all out; but just at this moment my friend, Prof. Chas. A. Budd, providentially called on me, and was immediately shown to the room. He declared that she was dead, and "laughed in his sleeve" at the idea of keeping up artificial respiration.

By this time I began to think of sending for an undertaker, (for she had come to me from a neighboring city for treatment,) but as a "drowning man clings to a straw," so I was eager to give her every possible chance, and asked Dr. Budd to suggest something, at the same time mentioning belladonna, when he said that atropine might be given hypodermically, if I wanted to do something, but as she was dead it would not bring her around. We resolved, however, to try it. By this time artificial respiration had been kept up for an hour and a half. One-sixtieth of a grain of the sulphate of atropia was injected, and in fifteen minutes she showed signs of life, the pupils began very slightly to dilate, and in ten minutes more she began to breathe, and the respiration rose to twelve per minute; in half an hour we repeated the dose, making in all one-thirtieth of a grain of atropine; and in about fifty minutes from the time of giving her the first injection, she returned to consciousness, and is living now, with a blank in her life of two and a half hours.

To Prof. Budd is due the credit of suggesting the remedy. —*N. Y. Medical Record.*

